# Case report: Giant meningioma of the left hemisphere

**DOI:** 10.3389/fonc.2024.1506297

**Published:** 2024-12-06

**Authors:** Junxiang Cui, Hu Sun, Shuo Sun, Hao Zhao, Yinghao Gu

**Affiliations:** ^1^ School of Clinical Medicine, Shandong Second Medical University, Weifang, Shandong, China; ^2^ Department of Neurosurgery, Zibo Central Hospital, Zibo, China

**Keywords:** meningioma, giant meningioma, sodium fluorescein, surgical operation, functional area

## Abstract

Meningiomas are some of the most prevalent primary brain tumors in adults, and are typically non-neuroglial in nature. A variety of symptoms may be observed, including headaches, fluctuations in mental status, ataxia, muscle weakness, nausea and vomiting, seizures, visual changes, speech disorders, and sensory abnormalities. The World Health Organization (WHO) has a grading system for meningiomas based on histological criteria, which is as follows: Grade 1 meningiomas are considered benign; Grade 2 meningiomas have a moderately aggressive nature and usually present with histological atypia; and Grade 3 meningiomas exhibit aggressive malignant behavior. Grade 3 meningiomas are distinguished by aberrant and accelerated cellular proliferation, which increases the probability of invasion and recurrence within the central nervous system relative to the other grades. Malignant meningiomas are further classified by tumor size. For example, WHO grade 3 meningiomas with diameter >5 cm are designated giant meningiomas. Giant meningiomas are complicated by their potential for compression of the brain tissue, which can lead to increased intracranial pressure and hemodynamic changes. In many cases, these changes induce vasogenic edema in the adjacent brain tissue. This article details a rare case of rapidly growing atypical giant meningioma that progressed to an anterior-posterior diameter of 13 cm within 3 years, occupying the majority of the left hemisphere of the brain and encroaching upon the right intracranial structures. Through recent advances in medical diagnostics and heightened public awareness of health issues, cases with such large meningiomas have become exceedingly rare. Fortunately, the tumor in the present case was successfully resected using advanced surgical techniques that employed microscopy in conjunction with sodium fluorescein, resulting in complete removal of the tumor and restoration of the patient’s muscle strength postoperatively. The value of fluorescence-guided surgery in this type of procedure is support in the present case report.

## Introduction

Meningiomas are the most prevalent intracranial neoplasms, accounting for approximately 15% to 20% of all such cases (1, 2). They are relatively slow-growing extra-axial tumors (3, 4), most frequently observed in the convex, parasagittal, or sickle regions of the skull, the pterygoid wings, the saddle nodes, and the posterior cranial fossa. Based on their slow growth rate (5, 6), meningiomas frequently remain undetected until they have reached a size that causes clinical symptoms, particularly when they are located in the “silent areas” of the brain. Meningiomas may be of considerable size (>3 cm) or very large (>5 cm) at the time of diagnosis (7, 8). Giant meningiomas can be distinguished from other types of meningiomas by three key characteristics: their large size, the fact that they can cause increased intracranial pressure, and their proximity to critical anatomical structures. These tumors are exceedingly rare and their characteristics frequently make them challenging for surgeons to excise completely (9). In the present case, the patient developed a large meningioma in the left hemisphere that grew to a diameter of 13 cm during a 3-year period. By employing sodium fluorescein and microscope-assisted techniques, we successfully achieved complete resection of the tumor. In the postoperative period, the patient’s muscle strength in the right upper extremity improved from grade 0 to grade III following a cerebral infarction, while the muscle strength in the right lower extremity improved from grade 0 to grade IV.

## Case presentation

**Figure 1:**
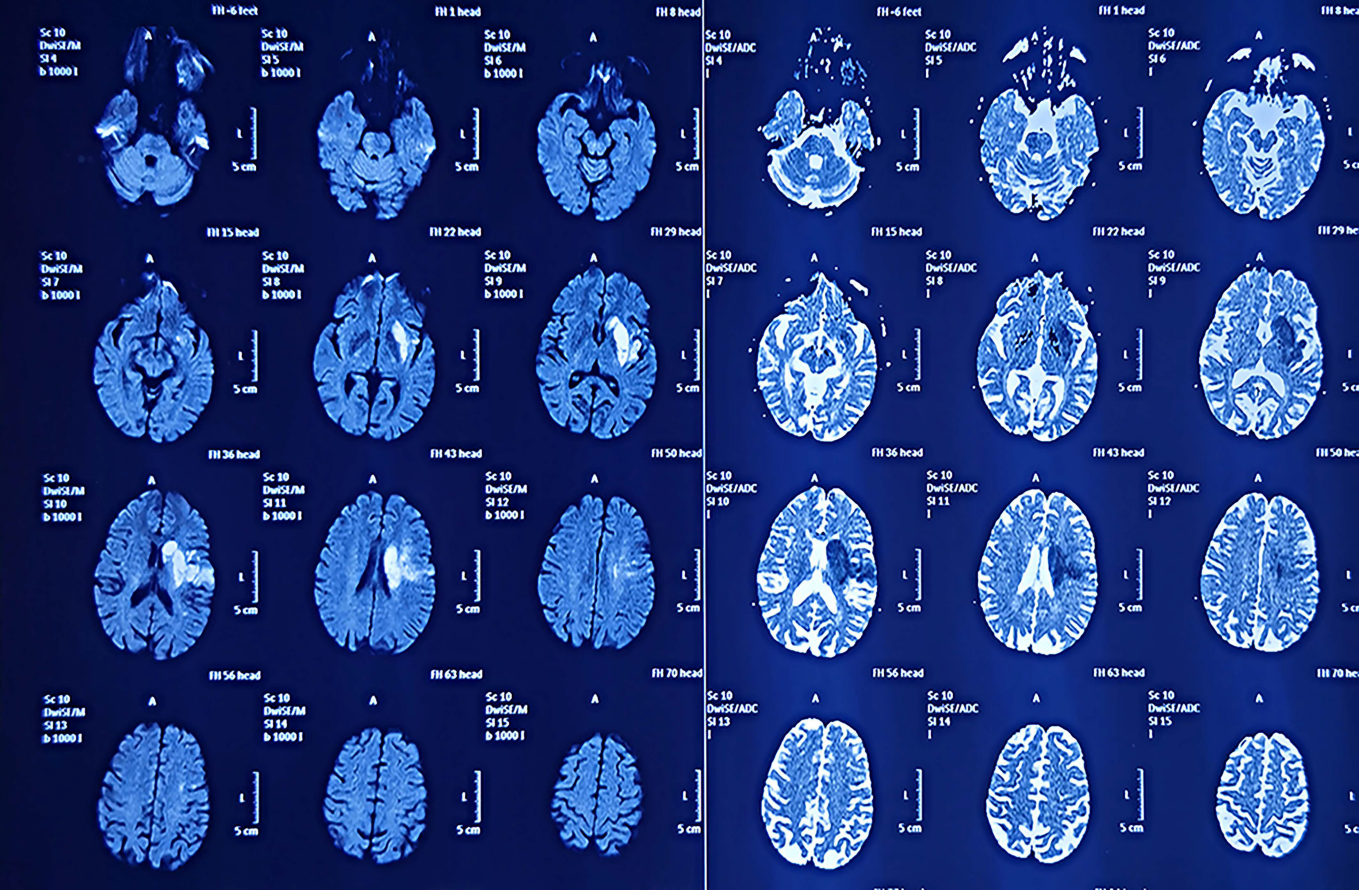
The patient’s MRI examination in 2021 showed a cerebral infarct but did not reveal the presence of a tumor.

**Figure 2:**
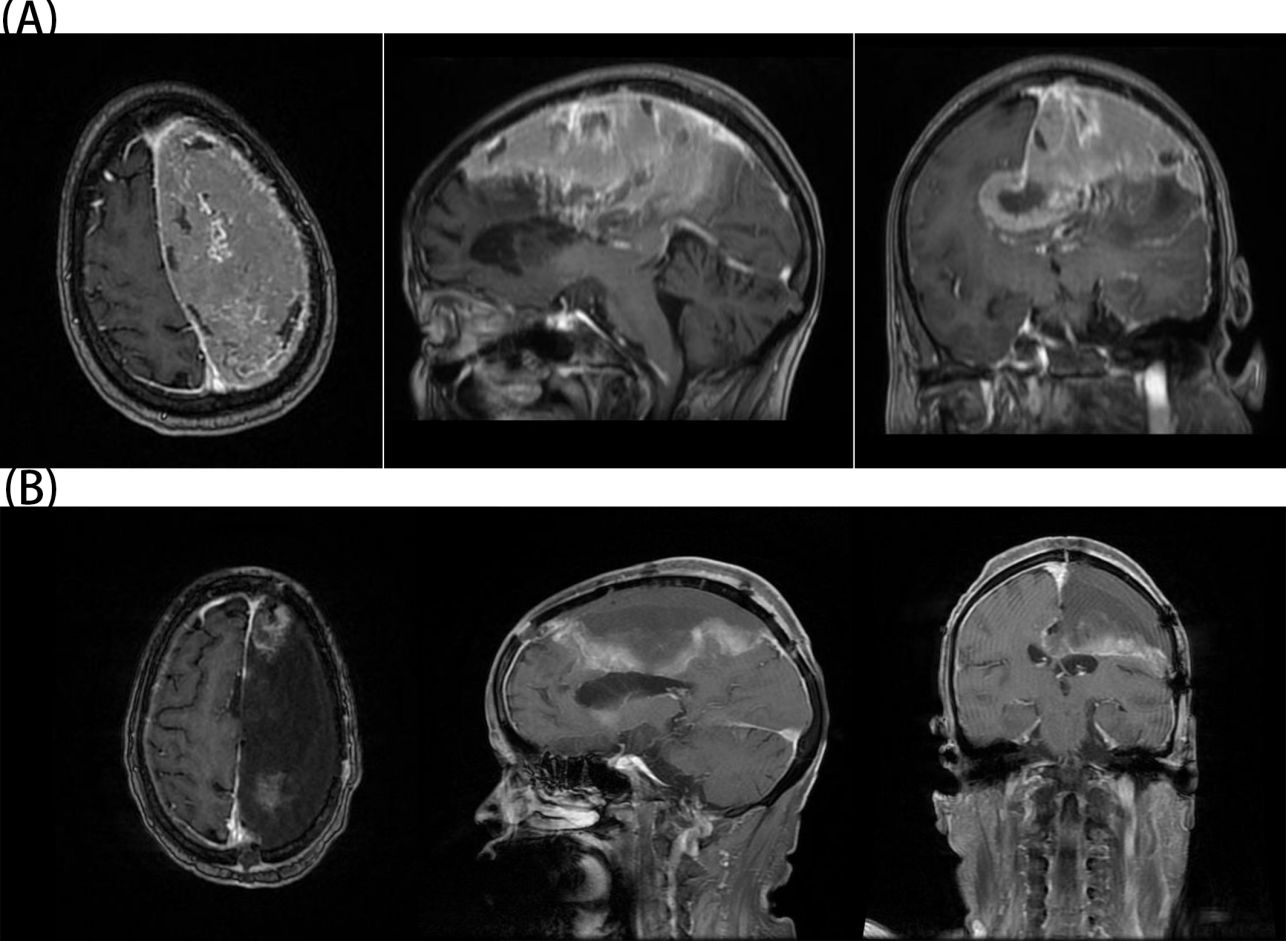
**(A)** The patient’s preoperative MRI showed a giant meningioma on the left side. **(B)** The patient’s postoperative MRI confirmed complete resection of the tumor.

**Figure 3:**
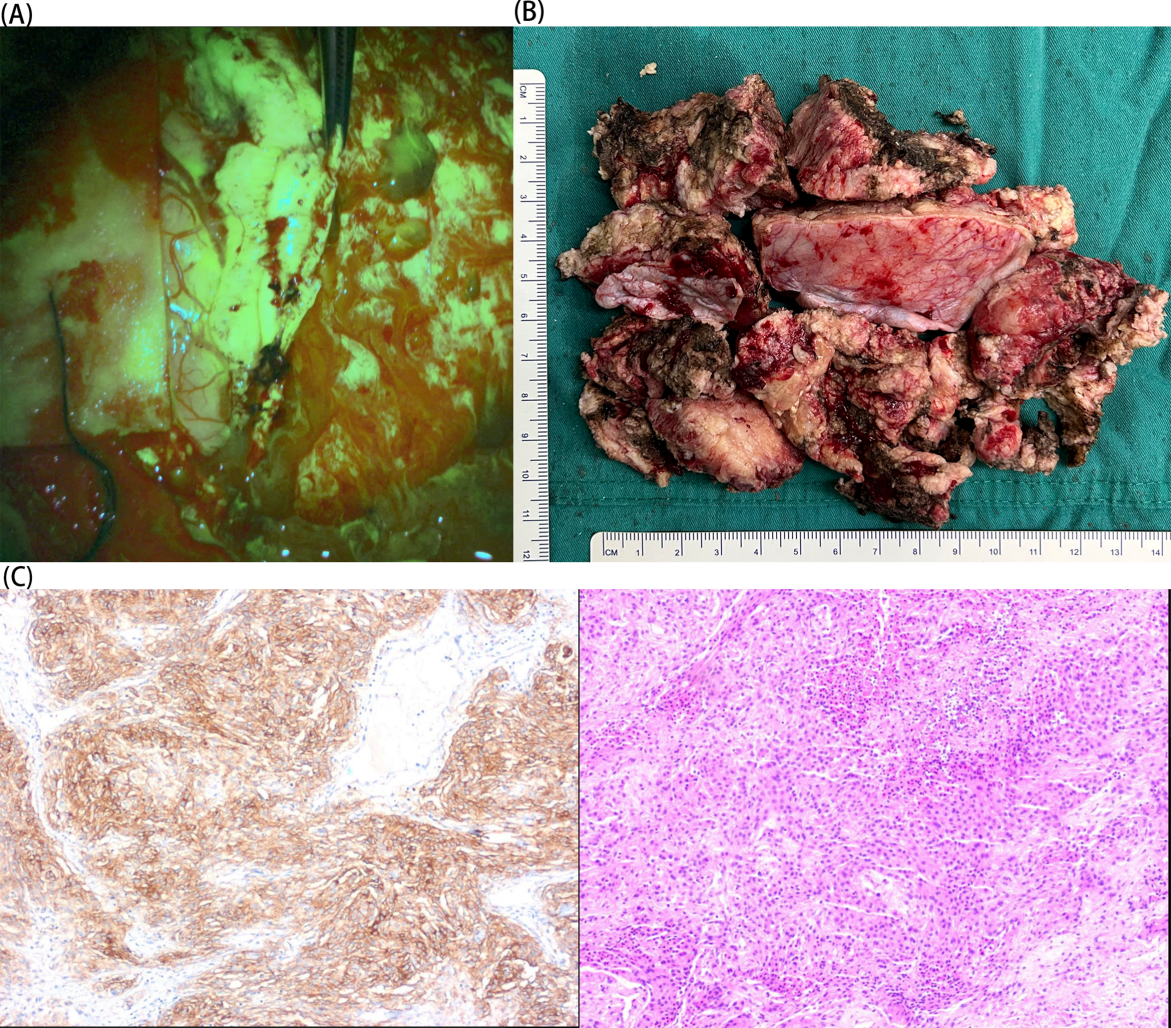
**(A)** Intraoperative sodium fluorescein visualization of the tumor. **(B)** Postoperative tumor specimen. **(C)** The patient’s histological findings (immunohistochemistry and HE: 10×magnification). The immunohistochemistry findings were:SSTR2,(+); vimentin, (+); S-100, (+); EMA, partial (+); GFAP, (−); ER, (−); PR, (−); AR, (−); SMA, (−); desmin, (−); CD10, (−); CD34, (−); Olig-2, (−); IDH-1, scattered (+); CKAE1/AE3, (−); CK8/18, (−); CK5/6, (−); P53, (−); Ki-67, (+) S accounted for 5%–20%.

**Figure 4:**
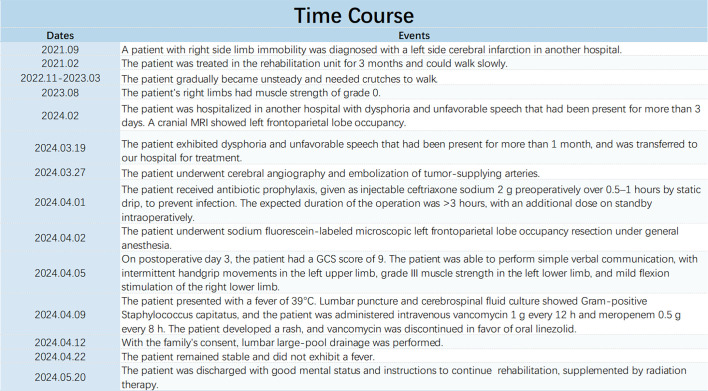
Patient Symptom Schedule.

A 77-year-old female patient was admitted to our hospital on 19 March 2024 with chief complaints of dysphoria and unfavorable speech that had been present for more than 1 month. Although the symptoms were intermittent, they were persistently worsening and accompanied by occasional headaches. The patient had a history of a cerebral infarction. A cranial MRI conducted in 2021 (**Figure 1**) did not reveal any obvious signs of an intracranial tumor. On admission, a physical examination revealed Glasgow Coma Scale (GCS) score of 11, incomplete motor aphasia, bilateral ocular collapse, unresisted neck, voluntary movement of the left limbs, and decreased muscle tone in the right limbs, with a muscle strength of grade 0. Laboratory tests, including electrocardiogram and routine blood and urine tests, yielded results within the normal ranges. A cranial MRI (**Figure 2A**) revealed a large occupying lesion (12.9×9.1×6.1 cm3) in the left cerebral hemisphere, invading the superior sagittal sinus in the right intracranial area. Multiple tortuous and increased vascular shadows were also observed in the vicinity of the lesion. A diffusion tensor imaging examination revealed that the projecting nerve bundles in the left frontal-parietal lobe and corpus callosum area showed partial atrophy compared with those on the contralateral side, with local irregularities suggestive of damage caused by tumor infiltration. The preoperative examination revealed a robust blood supply to the tumor, accompanied by diminished visualization of the left internal carotid artery system. Cerebral angiography was planned to identify any cerebral vascular lesions and the blood supply to the tumor. This was scheduled to be followed by a craniotomy at a later stage. The patient underwent the cerebral angiography under general anesthesia with embolization of the artery supplying blood to the tumor. Postoperative medications were administered to prevent complications, such as reduced intracranial pressure, and provide neuroprotection and symptomatic support. On postoperative day 6, the patient underwent fluorescein sodium labeling microscopy under general anesthesia to evaluate the extent of the resection in the left frontoparietal lobe. During this procedure, the tumor was visualized by sodium fluorescein via the frontotemporal-parietal approach over the midline (**Figure 3A**). A tumor measuring approximately 13×11×6 cm3 was successfully resected (**Figure 3B**), with the bilateral anterior cerebral arteries being well-protected and uninjured. Intraoperative dynamic monitoring of blood gas analysis revealed transfusion requirements for 11 units, 500 mL of plasma, and 10 units of cold precipitation. The patient’s intraoperative blood pressure and heart rate remained stable, the anesthesia was effective, and the patient was transferred back to the neurosurgical intensive care unit under anesthesia. The postoperative pathological examination (**Figure 3C**) revealed a giant atypical meningioma (WHO grade 2). A review of the postoperative cranial MRI revealed complete resection of the tumor (**Figure 2B**). On postoperative day 3, the patient had a GCS score of 9 and was able to perform simple verbal communication, with intermittent handshake movements in the left upper limb, grade III muscle strength in the left lower limb, and stimulation of the right lower limb with slight flexion. On postoperative day 7, the patient developed a fever that reached 39°C. A lumbar puncture was performed, and the cerebrospinal fluid culture revealed staphylococcus capitatus, indicating an intracranial infection. Meanwhile, the lung infection showed signs of worsening, and the patient was initiated on an escalating antibiotic regimen, comprising intravenous vancomycin 1 g every 12 hours and meropenem 0.5 g every 8 hours. Subsequently, the patient exhibited a rash, prompting the cessation of vancomycin and initiation of oral linezolid therapy. On postoperative day 12, the patient’s condition was characterized by severe intermittent fever, lethargy, and grade IV muscle strength in the left limbs. The left upper limb exhibited partial compliance with movement. The muscle strength of the right upper limb was classified as grade I, while that of the right lower limb was classified as grade III. After receiving the family’s consent, lumbar large-pool tube drainage was initiated. By postoperative day 20, the patient’s condition had stabilized, with no further fever and a smooth course of lumbar large-pool drainage. By postoperative day 48, the patient had made a full recovery. Her mental status was clear, her spirits were high, and her GCS score was 15. The muscle strength of the left limbs was classified as grade V-, the muscle strength of the right upper limb was classified as grade III (her muscle strength after the previous cerebral infarction was classified as grade III), and the muscle strength of the right lower limb was classified as grade IV. The patient had a right Babinski sign of (±). The patient (**Figure 4**) was discharged with instructions to continue rehabilitation, supplemented by radiation therapy.

## Discussion

Meningiomas comprise 15% to 20% of intracranial tumors (1, 2). The majority of these tumors are slow-growing and benign, while the remainder are aggressive or truly malignant. The tumors are typically situated in the subdural space and represent the most prevalent non-glial primary tumors within the skull. It is crucial to acknowledge that all brain tumors, irrespective of their pathological classification, have the potential to elicit severe or even fatal symptoms due to their mass effect, a phenomenon exemplified by meningiomas. Meningiomas typically grow relatively slowly, with an average growth rate of approximately 2.41 mm per year (5, 6). Consequently, meningiomas rarely cause clinical symptoms in their early stages. However, as time progresses, these tumors increase in size and begin to cause a range of different symptoms. A standard definition for the diameter indicating a giant meningioma remains to be established within the academic community. Definitions vary, with some defining diameters exceeding 4.5 cm, others defining diameters exceeding 5 cm, 6 cm, or 7 cm (7, 8, 10–14), and the majority of the literature defining diameters exceeding 5 cm as giant meningiomas (9). In 1950, White et al. (15) reported a meningioma weighing 1,353 g. In 1971, Rao et al. (16) reported a meningioma weighing 1,890 g. In 1982, Cech et al. (17) reported a meningioma with a maximum diameter of 22 cm. Finally, in 2002, Gutteridge and Wallace (18) reported a meningioma with a maximum diameter of >10 cm. A review of the literature reveals that meningiomas with very large diameters or large weights have been observed in conjunction with cranial lesions or significant extracranial masses. In the present case, the meningioma was entirely confined to the skull, resulting in significant compression of the brain tissue and a grade 0 muscle strength rating for the right limbs. Furthermore, the patient’s meningioma, which was absent on the previous MRI, developed and grew to a diameter of 13 cm in only 3 years, inconsistent with the conventional notion of a slow-growing meningioma. Nevertheless, the precise biological mechanism by which meningiomas attain such enormous sizes remains unclear. Ultimately, the tumor in the present case was completely resected using microscopy in conjunction with sodium fluorescein. Following the procedure, the patient’s muscle strength in the right upper limb was restored from grade 0 to grade III, while that in the right lower limb was restored from grade 0 to grade IV, indicating significant improvement. In the context of meningioma resection, identifying the caudal border between the meningioma and the surrounding dura mater represents a significant challenge. Sodium fluorescein, a fluorescent agent with an analogous mechanism of action to gadolinium, a contrast-enhancing substance utilized in magnetic resonance imaging, is capable of accumulating in regions where the blood–brain barrier is compromised, particularly in the area surrounding a tumor (19). By precisely controlling the injection time, it is possible to ensure that sufficient quantities of sodium fluorescein are flushed out of healthy areas while being retained in areas with an altered blood–brain barrier (20). This provides real-time fluorescence contrast during surgery, enabling neurosurgeons to identify tumor areas with greater clarity. The technique can also mitigate the shortcomings of conventional neuronavigation techniques, such as brain displacement or localization inaccuracies, and allow visualization of the contrast-enhanced tumor regions in real time (21–25). In a study involving 30 patients with newly diagnosed or recurrent meningiomas, 88% of the tumors exhibited homogeneous diffuse enhancement with sodium fluorescein, and the resection rate was 87%. The present findings also indicate that fluorescence-guided neurosurgery may be a promising technique for extending the resection of brain tumors. The use of sodium fluorescein as an alternative to 5-aminolevulinic acid addresses some of the limitations associated with that reagent (26). Studies have demonstrated that sodium fluorescein can effectively delineate adjacent vascular and neural structures during meningioma surgery, facilitating separation of the tumor from the brain tissue. The technique enhances surgical safety, facilitates the resection of complex vascularized meningiomas, and provides unique advantages for the visualization of hidden vascular structures (27, 28). In cases where meningiomas have a large blood supply, preoperative embolization of the dural arteries may facilitate surgical resection and reduce blood loss and complications (29–36). Nevertheless, preoperative embolization remains a topic of contention, because it has the potential to cause complications such as edema, hemorrhage, stroke, and cerebral nerve palsy (37–39). These risks are more prevalent in cases with large, highly vascularized meningiomas (40, 41), where embolization remains a viable option because the supplying artery is challenging to access. Therefore, the elevated risk of cerebral edema, hemorrhage, and suboptimal discharge outcomes is not unexpected. It has been reported (42) that embolization may have a negative impact on WHO grade 2/3 tumors, although the findings may have been affected by bias stemming from the fact that these tumors are large, have abundant blood flow, are challenging to resect, and are more likely to undergo embolization. Conversely, it has been proposed that embolization may diminish the likelihood of tumor recurrence and could be a valuable alternative for patients at elevated risk for surgical intervention (33, 40, 43–45). Furthermore, this fluorescence-guided surgical technique is particularly beneficial for patients whose tumors are located in nonverbal, sensory, motor, and cognitive regions (e.g., temporal and occipital lobes) and does not increase the incidence of postoperative complications. Moreover, the technique can minimize the probability of postoperative recurrence and does not impose an additional financial burden on the patient. Neurosurgery central nervous system infections are a group of infections that occur within the skull and spinal canal with an incidence of 4.6%–25% (46). The pathogenic organisms include gram-negative bacteria, gram-positive bacteria, and fungi, with the former two being predominant (47). In the event of a suspected central nervous system infection, it is imperative that samples such as cerebrospinal fluid are collected for testing prior to the administration of any antimicrobial agents. Furthermore, empirical antimicrobial therapy should be initiated without delay (48, 49). Antimicrobials are the preferred treatment option over fungicides that can readily cross the blood–brain barrier, such as ceftriaxone, cefotaxime, meropenem, and vancomycin. Infections caused by methicillin-susceptible *Staphylococcus aureus* can be treated with ampicillin/sulbactam. Despite its unfavorable pharmacokinetic and pharmacodynamic profile, vancomycin is currently recommended as a first-line agent for methicillin-resistant *S. aureus* infections. For the treatment of third-generation cephalosporin-susceptible Gram-negative bacillus infections, ceftriaxone or cefotaxime is recommended; for *Pseudomonas* spp. strains, cefepime, ceftazidime, or meropenem is recommended. It is further recommended that preoperative antimicrobial prophylaxis should target the bacteria most likely to cause an infection, rather than killing all organisms (50). It is also important to note that routine and continuous prophylactic use of antimicrobials does not reduce the incidence of intracranial infections; rather, it increases the risk of drug-resistant strains of bacteria (51).

## Conclusion

The advent of advanced medical imaging techniques has enabled early diagnosis of meningiomas, prior to the onset of symptoms. Nevertheless, some patients may not be diagnosed until their tumor is at an advanced stage, by which time their symptoms may have persisted for years or have been previously misdiagnosed as other conditions. The risk of complete resection is elevated for giant meningiomas, because they frequently infiltrate crucial regions of the brain and are intricately linked to vital neurovascular structures. The surgical procedures employed to remove these tumors are particularly challenging for several reasons, including limited visual field, increased brain edema, high tumor vascularization, and potential need for extensive craniotomies. Furthermore, patients with larger meningiomas exhibit a higher incidence of peritumoral edema than patients with smaller tumors. The utilization of fluorescence-guided surgery in meningioma surgery can facilitate dissection of the tumor interface through clear visualization of adjacent vascular and neural structures. In critical areas of the brain, where cerebral edema is exacerbated and tumor necrosis is poorly demarcated from the cortex, the tumor can be resected as much as possible while protecting the blood vessels and nerves. The resection can also be combined with preoperative embolization in patients with large tumors or meningiomas with abundant vascularization. The present case corroborates the value of fluorescence-guided surgery in such procedures, illustrating its benefits in maximizing tumor resection while safeguarding the normal brain tissue.

## Data Availability

The original contributions presented in the study are included in the article/supplementary material. Further inquiries can be directed to the corresponding author.
